# Asymmetric Cryo-EM Structure of Anthrax Toxin Protective Antigen Pore with Lethal Factor N-Terminal Domain

**DOI:** 10.3390/toxins9100298

**Published:** 2017-09-22

**Authors:** Alexandra J. Machen, Narahari Akkaladevi, Caleb Trecazzi, Pierce T. O’Neil, Srayanta Mukherjee, Yifei Qi, Rebecca Dillard, Wonpil Im, Edward P. Gogol, Tommi A. White, Mark T. Fisher

**Affiliations:** 1 Department of Biochemistry and Molecular Biology, University of Kansas Medical Center, Kansas City, KS 66160, USA; amachen@kumc.edu (A.J.M.); ctrecazzi@kumc.edu (C.T.); poneil@kumc.edu (P.T.O.); srayanta@gmail.com (S.M.); 2 Department of Biochemistry, University of Missouri, Columbia, MO 65211, USA; akkaladevin@missouri.edu (N.A.); whiteto@missouri.edu (T.A.W.); 3 Departments of Biological Sciences and Bioengineering, Lehigh University, Bethlehem, PA 18015, USA; yfqi@chem.ecnu.edu.cn (Y.Q.); woi216@lehigh.edu (W.I.); 4 National Center for Macromolecular Imaging, Baylor University, Houston TX 77030, USA; rdillard@gmail.com; 5 Department of Biological Sciences, University of Missouri-Kansas City, Kansas City, MO 64110, USA; gogole@umkc.edu; 6 Electron Microscopy Core Facility, University of Missouri, Columbia, MO 65211, USA

**Keywords:** anthrax toxin, lethal factor, protective antigen, pore formation, translocation, nanodisc, cryo-EM, cryoSPARC

## Abstract

The anthrax lethal toxin consists of protective antigen (PA) and lethal factor (LF). Understanding both the PA pore formation and LF translocation through the PA pore is crucial to mitigating and perhaps preventing anthrax disease. To better understand the interactions of the LF-PA engagement complex, the structure of the LF_N_-bound PA pore solubilized by a lipid nanodisc was examined using cryo-EM. CryoSPARC was used to rapidly sort particle populations of a heterogeneous sample preparation without imposing symmetry, resulting in a refined 17 Å PA pore structure with 3 LF_N_ bound. At pH 7.5, the contributions from the three unstructured LF_N_ lysine-rich tail regions do not occlude the Phe clamp opening. The open Phe clamp suggests that, in this translocation-compromised pH environment, the lysine-rich tails remain flexible and do not interact with the pore lumen region.

## 1. Introduction

The lethality of anthrax, a zoonotic disease and bioterrorism agent, is due to the anthrax toxin. This tripartite toxin consists of a protective antigen (PA), lethal factor (LF; a mitogen-activated protein kinase kinase protease), and edema factor (EF; an adenylate cyclase) [1]. After secretion from *Bacillus anthracis*, the 83 kDa PA (PA_83_) binds to its target host cell receptor, either capillary morphogenesis protein 2 (CMG2) or tumor endothelium marker-8 (TEM8) [1,2,3,4]. PA_83_ is cleaved by proteases, resulting in 20 kDa and 63 kDa fragments. PA_63_ then self-associates to form a heptameric PA prepore that can associate with up to three molecules of LF or EF [5]. Octameric PA prepores may also assemble in solution, governed by LF or EF binding to PA_63_ monomers clipped in solution [6]. Receptor-bound assembled complexes are endocytosed. As the endosome acidifies to pH 5.0 (late endosome), the receptor-bound PA prepore undergoes a conformational change into an extended β-barrel pore structure that penetrates the endosomal membrane. This newly-formed structure facilitates unfolding and translocation of the 90 kDa LF (or EF) enzyme across the pH gradient of the endosomal membrane through the narrow PA pore lumen in a pH-driven hypothesized Brownian ratchet mechanism [7]. Translocation of α-helical regions of LF are aided by the PA α-clamp [8,9]. LF translocation is gated by a ring of seven phenylalanine residues, termed the Phe clamp, located further down the PA pore lumen [10,11,12,13]. The directional translocation of LF depends on protonation of acidic residues, the electrostatic character of the PA pore lumen, and any residual positive charges on LF [14]. Subsequent deprotonation of the translocating peptide after passing through the Phe clamp prevents back transfer. Translocated LF refolds on the cytosolic side of the endosomal membrane, where it disrupts cell signaling by cleaving MAP kinase kinases, resulting in cell death [15].

Previously, the Krantz group published work on the large-scale rearrangement of LF that occurs upon binding to the PA prepore. Specifically, the N-terminal α-helix of LF moves away from the main body of LF and is resituated into a groove in the interior surface of the PA prepore cap, termed the α-clamp region [16]. This reposition is proposed to help funnel the N-termini of LF into the PA pore lumen. The narrowest part of the pore lumen is the Phe clamp [10,17]. PA F427, which forms the heptameric Phe clamp, is an essential residue that facilitates LF translocation [12,18]. Mutations in this residue (e.g., F427A) affect the kinetics of pore formation and translocation [19]. Interestingly, the loop containing F427 (2β_10_–2β_11_ loop) was suggested by Jiang et al. [17] to be involved in the first unfolding step of the pore formation mechanism. This mechanism is based on a comparisons of crystal structures of the oligomeric PA prepore [20] and the 2.9 Å cryo-EM pore structure [17]. The 2β_10_–2β_11_ loop also contains D426 which forms a conserved inter-subunit salt bridge with K397 in the PA pore [11]. These interactions orient F427 into its constricted Phe clamp formation, which is hypothesized as pi-stacking interactions between adjacent F427 residues [1,11]. The first step of this pore forming mechanism is based on the increased flexibility of the 2β_10_–2β_11_ loop in various prepore crystal structures. Early characterization of LF-PA interactions showed the N-terminal tail of LF interacts with the Phe clamp of the PA pore at pH 5.0, which has since been verified using cysteine cross-linking [12,13].

PA pore formation is necessary, but not sufficient, for lethality: LF must be translocated through the pore. Das and Krantz [9] recently proposed the Phe clamp region is dynamic and can undergo large-scale movements to momentarily increase the pore diameter from 6 Å to 10–12 Å. These movements could resemble transient open forms due to salt bridge formation between the acidic residues in the Phe clamp loop and an adjacent monomer [11]. This latter conformation (open Phe clamp loop) affects translocation rates since mutations that inhibit salt bridge formation impact translocation kinetics. In this particular model, Krantz also presented single-channel evidence that α-helical structures translocate more efficiently than extended β-sheet-like structures or unstructured polypeptides containing alternating L- and D-amino acids. Pentelute et al. [21] showed chirality is not important for translocation of the unstructured region of the N-terminal domain of LF (LF_N_) either, but this does not preclude the possibility that α-helical structures could be formed upon the electrostatic interaction between LF_N_ and the PA pore. This would have to include α-helices that are in the d chiral form since all L- or D-amino acid α-helical structures do not slow down translocation. It would then be of value to determine if the Phe clamp loop region becomes more structurally dynamic (loss of resolution) and/or adapts a more open configuration upon interaction with the single or multiple unstructured lysine-rich tails of bound lethal factor(s).

Understanding both pore formation and LF translocation is imperative in order to develop strategies that mitigate or prevent the formation of the anthrax toxin complex or inhibit the translocation mechanism. Inhibition of circulating anthrax toxins is crucial since the toxin components retain cell lethality even after the bacilli have been killed with antibiotics [1]. To better understand the interactions between LF_N_ and PA, the structure of the LF_N_-bound PA pore in a lipid membrane environment was examined using cryo-electron microscopy (cryo-EM).

## 2. Results

### 2.1. Cryo-EM Sample Preparation of PA Pore with Three LF_N_ Bound

With the recent publication of the cryo-EM PA pore structure at pH 5.0 [17], the logical, but challenging, next step in understanding anthrax toxin pore formation and translocation involves determining how bound LF influences the conformation of the PA pore. An atomic resolution structure of LF_N_-bound PA pore would give molecular insight into the nuances of this interaction. In order to solve the cryo-EM structure of the LF_N_-PA pore, several obstacles must be overcome, including the aggregation propensity of the pore, asymmetry of the LF_N_-PA complex, and orientational preferences of complexes on EM grids.

**Figure 1:**
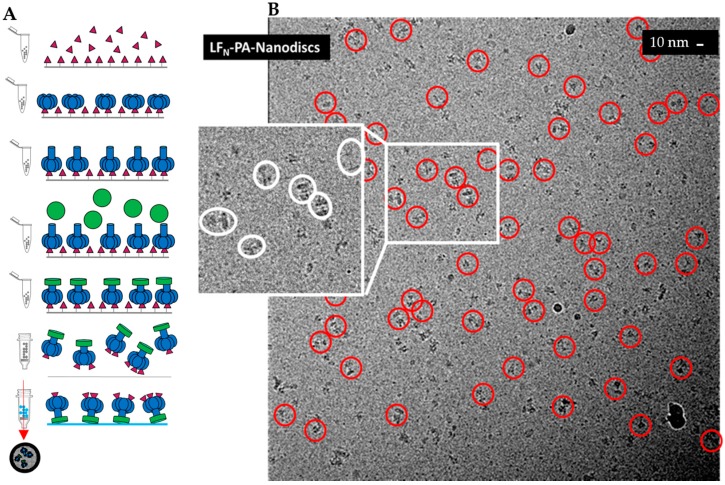
Sample preparation of 3LF_N_-PA-nanodiscs: (**A**) schematic of LF_N_ (magenta)-PA (blue)-nanodisc (green) complex formation with stepwise addition of LF_N_ and PA to thiol sepharose beads; and (**B**) a higher defocus representative field for high-contrast visualization. Individual LF_N_-PA-nanodisc complexes may be easily observed within this micrograph. Note the variable size of the nanodiscs in the insert.

We previously published a methodology to assemble LF_N_-PA pore complexes while avoiding aggregation by immobilizing PA pores before solubilizing the hydrophobic tip with lipid bilayer nanodiscs [22,23,24,25]. After immobilization, the PA prepores were transitioned into pores using a urea/37°C pulse methodology, exposing the aggregation-prone pore tip. The nanodisc formed around the hydrophobic pore tip while the complex was immobilized [22,23,25,26]. A schematic of this methodology is shown in Figure 1A. Our previous low-resolution LF_N_-PA-nanodisc structures were reconstructed from samples frozen on perforated carbon containing a thin carbon layer over holes [23]. There were a number of caveats limiting the structural analysis of that preparation. Most importantly, large diameter nanodiscs (approximately 400 Å) were generated and required the use of thicker ice. In addition, LF_N_-PA-nanodisc complexes interacted with the carbon layer, resulting in complexes preferring a side view orientation which displays the long axis of the heptameric PA pore rather than allowing for more diverse conformational orientations, including top views. Although these LF_N_-PA-nanodisc complexes were inherently structurally asymmetric (symmetry mismatch of seven PA subunits to a maximum of three LF_N_ bound), their structures were generated by imposing seven-fold symmetry, which resulted in smearing of the LF_N_-bound density. This coupled with the sample-induced constraints (thin carbon backing, thick ice, and Fresnel fringe effects for the sharp nanodisc protein interface) diminished the contrast of the protein. These constraints also interfered with the visualization of the PA β-barrel in the reconstruction.

To obtain a more concise LF_N_-PA-nanodisc complex structure, these sample preparation issues had to be overcome. For better contrast, samples were frozen on simple perforated carbon grids without a thin carbon layer in order to achieve greater orientational diversity and were imaged with a JEM 2200FS electron microscope (60,000× magnification) (JEOL, Peabody, MA, USA). A representative micrograph with high defocus for better contrast (for visualization, not reconstruction purposes) with individual particles highlighted with red circles is shown in Figure 1B. Low-dose, low-defocus conditions were used to collect images for 3D reconstruction. Notably, the nanodiscs for these samples were significantly smaller than the previous larger nanodisc samples. The nanodisc size was dependent on the length of time that LF_N_-PA-nanodisc complexes were immobilized as well as rotation of the sample tube. Under non-ideal conditions, the pre-nanodisc micelles may merge, generating larger nanodisc diameters. Interestingly, larger nanodiscs often resulted in multiple PA pore-inserted nanodisc complexes (e.g., sometimes four PA pores inserted into one large nanodisc). These larger nanodiscs were attributed to longer dialysis times that consistently resulted in merging of pre-nanodisc micelles. Reducing the time of incubation, ensuring adequate detergent dialysis with Bio-Beads, and constant rotation during formation yielded smaller nanodiscs within the expected diameter range (100–150 Å) containing a single PA pore (Figure 1B).

### 2.2. Single-Particle Analysis of LF_N_-PA-Nanodisc Complexes

**Figure 2:**
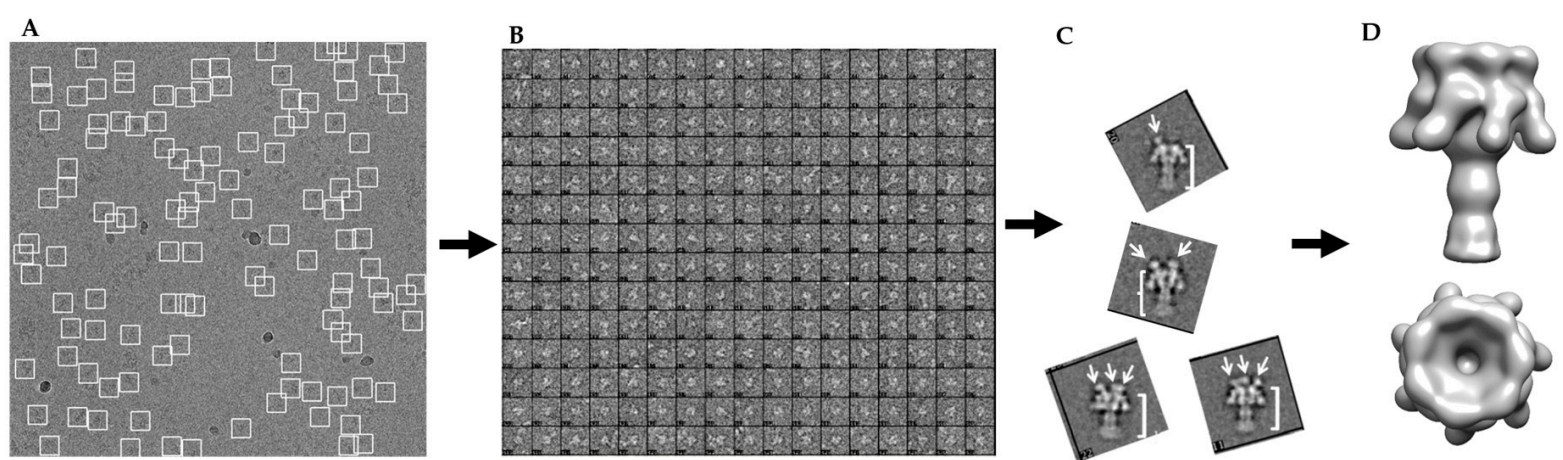
Schematic of data analysis of heterogeneous cryo-EM data: (**A**) sample micrograph field showing good ice and particle distribution; (**B**) an example of individual boxed particles from micrographs with phase inversion for contrast; (**C**) SPARX 2D class averages (side views) reveal heterogeneity of sample preparation with arrows indicating LF_N_ binding; and (**D**) 3D model of 3LF_N_-PA pore with C7 symmetry imposed smears LF_N_ density into a crown around the top of the pore.

Initial classification analysis using SPARX [27] revealed heterogeneity in the dataset with one, two, or three LF_N_ bound to PA pores (Figure 2A–C). The release of LF_N_-PA-nanodisc complexes from the bead surface into solution also resulted in the release of non-complexed LF_N_, which was then able to bind released complexes leading to particles with multiple binding events. This led to subsets of PA having one, two, or three LF_N_ bound. This inherent heterogeneity in LF_N_ binding stoichiometry made 3D reconstruction difficult. Initially, this limited particle dataset could only be used to obtain a model by imposing C7 symmetry during reconstruction using EMAN2.1 and RELION (Figure 2D).

**Figure 3:**
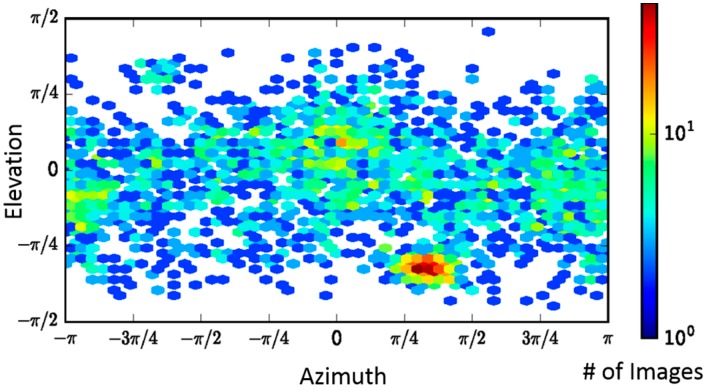
Direction distribution map of particles, analogous to an Euler angle map, showing the conformational coverage of LF_N_-PA-nanodiscs.

While PA alone has C7 symmetry, LF_N_-bound PA in a saturated (three LF_N_ bound) or sub-saturated binding ratio only possesses C1 symmetry. The recent successful high-resolution reconstruction of the PA pore at pH 5.0 by Jiang et al. [17] was accomplished using, primarily, top and side view orientations that were generated by taking advantage of a grid adherence platform. In that sample preparation, the prepore adhered to the carbon layer through its receptor binding interface and the pore transition was accomplished by adjusting the pH of the solution to pH 5.0. Since the pore itself has an axis of seven-fold symmetry, the variable positioning of the side views of the PA pore on the carbon layer were sufficient to cover most of the conformational space to obtain the first high-resolution structure (2.9 Å) of the anthrax toxin pore translocon [17]. With LF_N_-PA-nanodisc complexes, the nanodisc insertion procedure permits conformational diversity, which is critical for obtaining a structure without imposing sevenfold symmetry. A direction distribution map, analogous to an Euler angle map, confirmed the orientation of the LF_N_-PA-nanodisc particles was conformationally diverse (Figure 3).

It is important to note this diverse distribution is crucial for acquiring the asymmetric LF_N_-PA-nanodisc structures since the imposition of sevenfold symmetry during 3D reconstructions distorts the density of any bound LF_N_ (Figure 2D). CryoSPARC is well suited to obtain unbiased, reproducible, and reliable *ab initio* 3D models rapidly even when extensive sample heterogeneity is present [28,29]. For example, Ripstien et al. [30] reexamined their previous cryo-EM data of the *Thermus thermophiles* V/A-ATPase using cryoSPARC and were able to determine their ATPase sample was actually populated by multiple conformations that were previously unresolved, resulting in new mechanistic insights.

**Figure 4:**
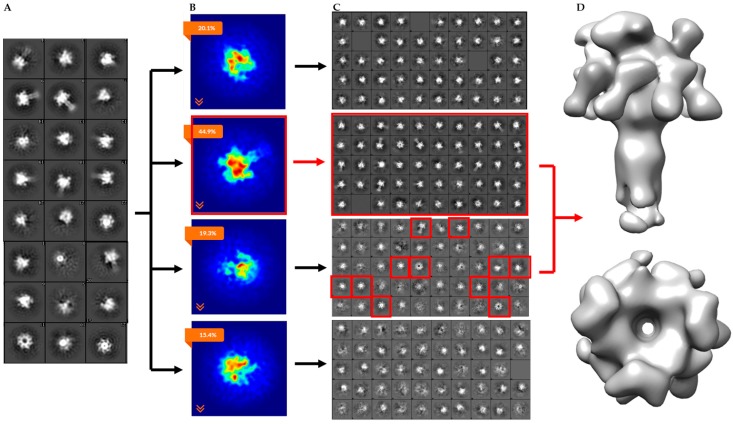
CryoSPARC data analysis flowchart of heterogeneous LF_N_-PA-nanodiscs with total computational time of 3.5 h from 2D averaging to refined model: (**A**) cryoSPARC 2D class averaging of 18,806 particles; (**B**) image projection of heterogeneous *ab initio* reconstruction with four groups, the largest group, with 44.9% of the particles, corresponds to 3LF_N_; (**C**) 2D class averages of each *ab initio* particle group; and (**D**) 17 Å model of 3LF_N_-PA generated from homogeneous refinement of the Group 2 *ab initio* model with particles from top and bottom 2D class averages highlighted in red.

To separate the heterogeneous LF_N_-PA-nanodisc particles, an initial 2D classification was performed on the 30,696 particles with removal of bad classes as determined by eye (Figure 4A). An *ab initio* classification with four groups was then performed on the remaining 18,806 good particles (Figure 4B). Four groups were chosen since two LF_N_ can bind to PA at neighboring binding sites or with an empty binding site between them resulting in 1LF_N_, 2_A_LF_N_, 2_B_LF_N_, or 3LF_N_ bound. Group 2 was the most highly populated group identified by the cryoSPARC stochastic gradient descent (SGD) *ab initio* model generation with three distinct and equal LF_N_ densities (Figure 4B). Further 2D classification was performed on all four groups to assess the quality of particles within each group (Figure 4C). Group 3 contained several highly-populated classes showing sharp sevenfold symmetric top and bottom views. Group 1 and 4 particles did not result in clear classes and were discarded (Figure 4C, top and bottom panels). Since the top and bottom view classes in Group 2 were underrepresented, all particles from Group 2 (4560) and particles from the good classes in Group 3 (1159) were combined. A homogeneous refinement was run with the Group 2 *ab initio* model with the combined good particle set (Figure 4D).

**Figure 5:**
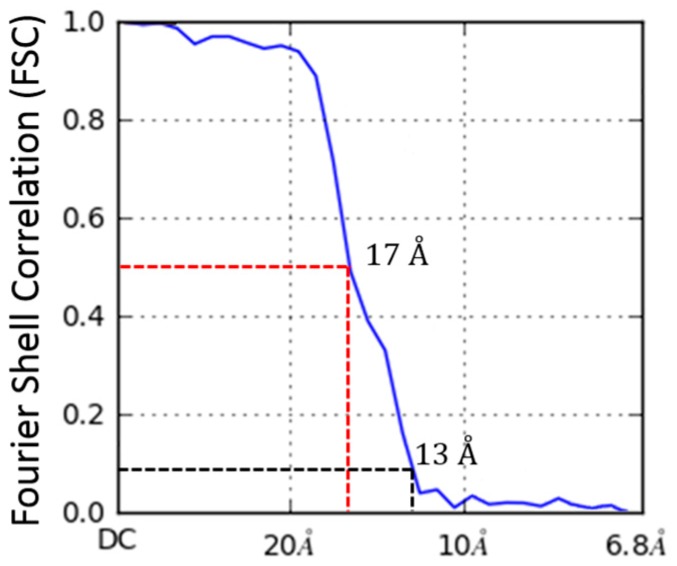
Fourier shell correlation (FSC) for 3LF_N_-PA-nanodiscs. Resolution estimated to be 17 Å based on FSC with a cutoff of 0.5. This conservative cutoff agrees with filtered models shown later in Figure 10.

The homogeneous refinement resulted in a 17 Å 3LF_N_-PA pore model from 5719 particles. Figure 5 shows the Fourier Shell Coefficient (FSC) used to calculate the resolution. This resulting reconstruction was not biased by outside models or symmetrization operations. The β-barrel pore of PA was not prominent in the *ab initio* model but became more apparent upon cryoSPARC refinement. The bulge in the β-barrel of the final model was also seen in the cryo-EM structure of the PA pore alone where this hydrophobic region of the outer barrel bound lipids, resulting in the accumulation of additional density [17]. As can be seen in the 2D classification (Figure 4C, second panel), side view images reveal variation either in nanodisc size or electron density. This resulted in a lack of nanodisc structure in the final electron density map. The irregular density at the bottom of the pore tip in the final structure can be attributed to either the presence of nanodisc or free lipid binding to exposed hydrophobic residues. As mentioned previously, the decrease in nanodisc density appears to be due to extended dialysis times during micelle to nanodisc collapse. The decreased nanodisc size did not diminish our ability to reconstruct LF_N_-PA pore complexes, particularly in the PA pore cap and the initial extension of the β-barrel.

### 2.3. Constructing Samples with Highly-Populated Singly-Bound LF_N_-PA for Cryo-EM

**Figure 6:**
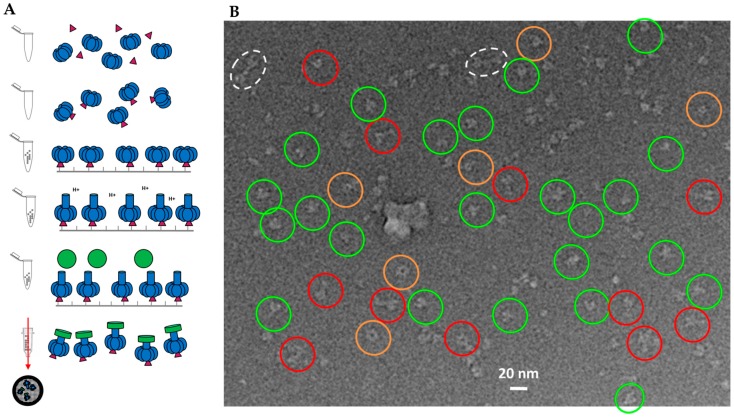
Sample preparation of 1LF_N_-PA-nanodiscs: (**A**) schematic of LF_N_ (magenta)-PA (blue)-nanodisc (green) complex formation with LF_N_ and PA prepore incubated prior to immobilization; and (**B**) representative cryo-EM image field of initial screening. Inverted contrast for visualization. Only select clear individual particles are noted (key: red—side views; green—various angle views; dotted white—double PA pores in a single nanodisc; orange—top and bottom views). Extra density from LF_N_ binding is occasionally observed, particularly in the side view orientations.

**Figure 7:**
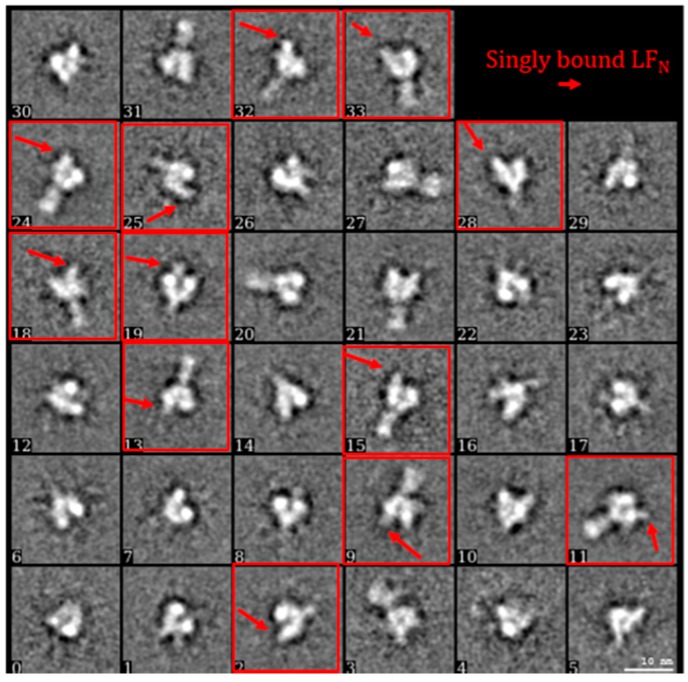
2D classification of approximately 1200 particles using SPARX confirmed singly-bound LF_N_ with examples of clear LF_N_ densities highlighted in red.

The heterogeneity of this sample preparation was due to the stepwise assembly of LF_N_-PA complexes, shown above in Figure 1A. LF_N_ was immobilized onto thiol sepharose beads, then PA prepore was added, binding to the LF_N_. The bulkiness of PA relative to LF_N_ blocked PA from binding to multiple LF_N_. After LF_N_-PA-nanodisc complexes were formed on the beads, they were released into solution. Any unbound LF_N_ was also released and, due to its high affinity for PA, bound to open binding sites of PA (Figure 1A). To obtain a larger, more homogeneous LF_N_-bound PA pore particle set, the protocol was modified by pre-incubating LF_N_ with PA prepore in a 1:2 ratio to ensure a higher population of singly-bound LF_N_-PA. A schematic of the updated protocol is shown in Figure 6A. As proof of principle for future structure determinations, an initial cryo-EM screen of complexes isolated with this new protocol was performed. Figure 6B shows a representative screening image collected on F30 twin TEM (FEI, Hillsboro, OR, USA) at 39,000 times nominal magnification and a pixel size of 3 Å on the specimen. 2D class averaging with SPARX (side views shown in Figure 7) showed the majority of the classified populations had single LF_N_ densities. As with all preparations using the immobilized construction of LF_N_-PA pore complexes, the elution volume is easily adjusted to obtain a sufficient concentration of particles on the grid for automated screening with a high-powered microscope with a direct electron detector.

### 2.4. Molecular Dynamics Flexible Fitting of 3LF_N_-PA Pore Model into the 17 Å Cryo-EM Density Map

**Figure 8:**
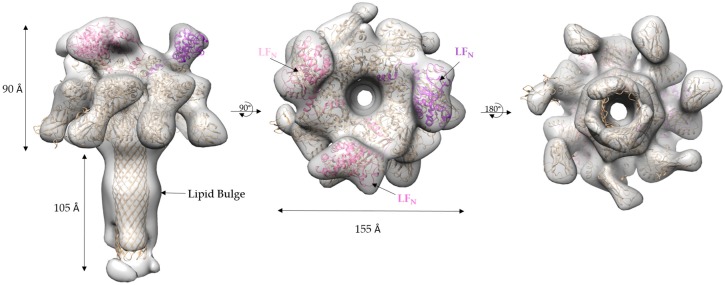
3LF_N_-PA cryo-EM density map (grey) with the ribbon structure MDFF-fitted 3LF_N_ (pink, magenta, and purple)-PA pore (gold): (**left**) side view; (**middle**) top view; and (**right**) bottom view.

**Figure 9:**
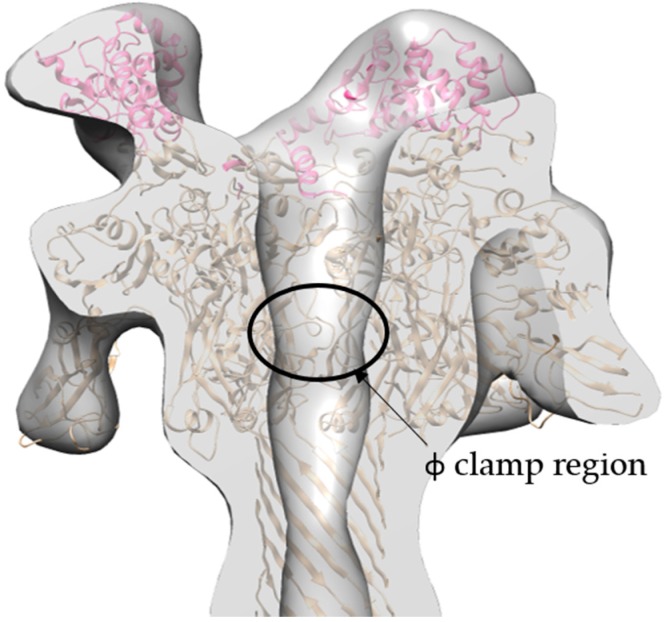
Cross-section of the side view cryo-EM density map (**grey**) with ribbon structure MDFF model of LF_N_ (**pink and magenta**) and PA (**gold**) reveals that the narrowing of the pore lumen in the density map is consistent with location of Phe clamp region.

The refined 17 Å cryo-EM model of 3LF_N_-PA-nanodisc generated by cryoSPARC has several interesting asymmetric features (Figure 8). As mentioned previously, there are three LF_N_ densities. A molecular dynamics flexible fitting (MDFF) of 3LF_N_-PA pore docked three LF_N_, in pink, magenta, and purple, in between subunit interfaces of PA, as was seen previously in the prepore crystal structure of 4LF_N_-8PA and confirmed by 15 Å cryo-EM structures using the complete LF-PA prepore structure [16,20]. Previous work has shown the N-terminal tail of LF_N_ feeds into the pore lumen and interacts with the Phe clamp. A cross-section of the model, shown in Figure 9, reveals the narrowing of the pore lumen is consistent with the positioning of the Phe clamp region in the MDFF model. Curiously, this pH 7.5 low-resolution triply-bound LF_N_-PA pore structure shows an open pore region, in contrast to the closed densities observed for the previous lower-resolution, seven-fold symmetrized structures [22].

**Figure 10:**
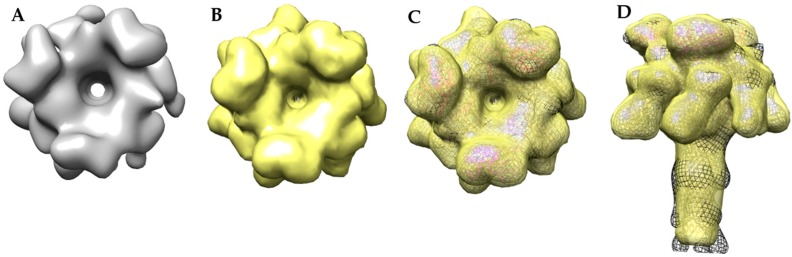
Comparison of the cryo-EM and MDFF models show similar topology of the LF_N_ bean shape and PA cap protrusion: (**A**) top view of the 17 Å cryo-EM map; (**B**) top view of the MDFF atomic resolution model filtered to 17 Å; (**C**) top view overlay of the 17 Å cryo-EM model (black mesh), the MDFF model filtered to 17 Å (yellow), and the MDFF ribbon model (PA in gold, LF_N_ in magenta); (**D**) side view overlay of the cryo-EM model (black mesh), the MDFF model filtered to 17 Å (yellow), and the MDFF ribbon model (PA in gold, LF_N_ in magenta).

A comparison of the MDFF atomic structure filtered to 17 Å with the 17 Å cryo-EM-derived 3LF_N_-PA pore structure showed surface details that were visually indistinguishable (Figure 10). For example, the top view of the cryo-EM 3LF_N_-PA structure showed LF_N_ has a distinctive bean shape (Figure 10A). A top view of the space filled PDB structure of LF_N_ bound to the prepore structure also had this same characteristic shape [16]. A small protrusion from the PA pore cap where LF_N_ is absent was also present in both models. Unlike the MDFF structure, the domain 4 regions of the cryo-EM derived structure are not equal in density, suggesting that these regions are dynamic structures as was previously observed by Jiang et al. [17]. It is also important to note that not all surface regions in the cryo-EM reconstruction are filled by MDFF analysis. For example, the β-barrel bulge that is due to lipid binding is not revealed in the fit structure since such a bulge in the highly-stable β-barrel is energetically restrictive.

## 3. Discussion

Atomic resolution cryo-EM is a rapidly evolving structural method that can be applied to examine the atomic consequences of LF_N_ interactions with the PA pore. The ability to generate soluble, lipid-stabilized LF_N_-PA pore structures, even in this low resolution model, is the critical, important first step in demonstrating that we can obtain structural snapshots of this complex.

### 3.1. Sample Preparation of Highly Pure Complexes

One of the main thrusts of this work has been to demonstrate that we can routinely obtain highly-pure engagement complexes (multiply- or singly-bound LF_N_) using an immobilization bead-based protocol and nanodisc technology without using columns to purify the final complexes [23,25] and minimizing detergent influences on structure [31,32]. Even at 17 Å resolution, the variability of the domain 4 densities for the LF_N_-PA pore indicates this region is intrinsically flexible [17], ruling out the possibility that this flexibility is due to grid adherence constraints. Although it is possible the insertion of the tip region into an authentic lipid bilayer (e.g., a nanodisc) may result in more ordered structures, better nanodisc resolution is required to make this assessment [33,34,35]. Previously, protein-bilayer interactions in nanodiscs have been noted to result in extended β-barrel protein structures (approximately two residues per strand) compared with detergent-solubilized structures [35].

### 3.2. Initial Cryo-EM Model of 3LF_N_-PA Pore

The cryo-EM density map structure was created without imposing symmetry or biasing towards an initial input model using the cryoSPARC *ab initio* reconstruction and subsequent refinement procedures. This 17 Å 3LF_N_-PA pore model showed three distinct LF_N_ densities. In agreement with what was observed previously, the LF_N_ densities are positioned between two protomer interfaces of the PA pore [16,20]. The main contact points are on the crest of the pore and in the α-clamp. Only three LF_N_ are able to bind to a heptameric pore, leaving one protomer without any direct LF_N_ contacts.

A cross-section through the EM density map showed the location of the pore opening complete with the narrowing of the pore lumen. An MDFF fit starting from the atomic resolution pore structure with LF_N_ bound positions this narrowing region with the Phe clamp loop region and preserves the opening at the Phe clamp annulus. While the number of particles and subsequent resolution of this current cryo-EM density map do not allow us to definitively define structural details of the pore lumen, it would be of interest to determine if the pore remains in a more open configuration at pH 7.5 when one or three LF monomers are bound. This further highlights the need to obtain high-resolution structures of the PA pore with one or more LF bound to determine if the Phe clamp region remains more open under these conditions. As mentioned previously, the presence of interfering electrostatic interactions appears to lead to a more open pore structure. Notably, this open pore diameter has been suggested by Das and Krantz to be necessary in order to accommodate α-helical regions during translocation at pH 5.0. These atomic resolution structures will be key to determining if varying ratios of LF bound (i.e., one vs. three) induces significant structural asymmetry (variable positioning of the Phe clamp) or concerted symmetry (all open) on the PA pore structure.

It is not uncommon to observe both small- and large-scale symmetry breakage of ordered oligomers induced by protein-protein interactions. For example, structures of protein substrate and nucleotide interactions with GroEL, a tetradecameric ring chaperonin protein, show very discernable asymmetric adjustments due to protein substrate interactions [36,37], as well as ATP binding and hydrolysis [38]. A more dramatic demonstration for ligand-induced distortion of symmetry is observed for the ATP bound vs. ADP bound ATPase unfolding machinery of the valosin-containing protein-like ATPase (VAT) recently resolved by cryo-EM [39]. In this instance, the hexameric structure was dramatically distorted in the presence of ADP and appeared to coincide with its ATP/ADP conformational switching mechanism to provide a conformational platform that unfolds proteins prior to degradation.

It would be of great interest to compare singly bound and multiply bound LF_N_-PA pore structures in different pH conditions in order to discern any distinct structural differences that may result from being in various pH environments. Observing these different states of the engagement complex (pH 5.0 vs. pH 7.5, 1 LF_N_ vs. 3 LF_N_) would be useful in determining the position of the Phe clamp loop region and potentially defining unstructured regions of the LF_N_ that may become structured upon binding to the pore prior to translocation at pH 5.0. There are existing crosslinking studies by the Collier group indicating this interaction is present at pH 5.0 [13]. Thus, there is precedence for this interaction and those cryo-EM structure collection experiments at pH 5.0 are currently underway. In all cases, given the intrinsic stability of the extended β-barrel at pH 5.0 and pH 7.0, it is highly unlikely that the β-barrel region will be structurally altered when LF_N_ binds to the PA pore cap region. Rather, the more flexible parts of the PA pore (i.e., the cap region, Phe clamp region, etc.) will be highly susceptible to LF_N_-induced conformational changes. How LF structurally impacts translocation and pore formation may be manifested through long range allosteric affects.

## 4. Conclusions

Understanding both PA pore formation and LF translocation through the PA pore is crucial to mitigating, and perhaps preventing, anthrax disease. To better understand the interactions between LF_N_ and the PA pore, the structure of LF_N_-bound PA pore was examined using cryo-EM. The 17 Å structure of PA pore with 3 LF_N_ bound was the result of pore immobilization, nanodisc solubilization, *ab initio* modeling, and refinement. In this pH 7.5 structure, the contributions from the three unstructured LF_N_ lysine-rich tail regions do not occlude the Phe clamp opening, indicating these flexible tails remain unstructured and unresolved. The next structures to examine are the LF_N_-PA pore complexes at pH 5.0 to determine if the unstructured LF N-terminal tails interact with the Phe clamp.

## 5. Materials and Methods

### 5.1. Protein Expression and Purification

Recombinant wild-type (WT) PA was expressed in the periplasm of *Escherichia coli* BL21 (DE3) and purified by anion exchange chromatography [40] after activation of PA with trypsin [41]. QuikChange site-directed mutagenesis (Stratagene) was used to introduce mutations into the plasmid (pET SUMO (Invitrogen)) encoding a truncated recombinant portion of lethal factor. LF_N_ E126C and was expressed as His_6_-SUMO-LF_N_, which was later cleaved by SUMO (small ubiquitin-related modifier) protease, revealing the native LF_N_ E126C N-terminus [41]. Membrane scaffold protein 1D1 (MSP1D1) was expressed from the pMSP1D1 plasmid (AddGene) with an N-terminal His-tag and was purified by immobilized Ni-NTA affinity chromatography as previously described [42].

### 5.2. Formation of LF_N_-PA-Nanodisc Complexes

Heterogeneous LF_N_-PA-nanodisc complexes were formed and purified as previously described [22,25]. In brief, E126C LF_N_ was immobilized by coupling E126C LF_N_ to activated thiol sepharose 4B beads (GE Healthcare Bio-Sciences, Pittsburgh, PA, USA) in Assembly Buffer (50 mM Tris, 50 mM NaCl, pH 7.5) at 4 °C for 12 h. One hundred microliters (100 μL) of 0.2 μM heptameric WT PA prepore was then added to 50 μL of LF_N_ bead slurry. Beads were washed three times with Assembly Buffer to remove any unbound PA prepores. The immobilized LF_N_-PA prepore complexes were then incubated in 1 M urea (Thermo Fisher Scientific, Waltham, MA, USA) at 37 °C for 5 min to transition the PA prepores to pores. After three more washes with Assembly Buffer, pre-nanodisc micelles (2.5 μM MSP1D1, 162.5 μM 1-palmitoyl-2-oleoyl-sn-glycero-3-phosphocholine (POPC) (Avanti, Alabaster, AL, USA) in 25 mM Na-cholate (Sigma-Aldrich, St. Louis, MO, USA), 50 mM Tris, and 50 mM NaCl) were added and bound to the aggregation-prone hydrophobic transmembrane β-barrel of PA. The micelles were collapsed into nanodiscs by removing Na-cholate using dialysis with Bio-Beads (BIO RAD, Hercules, CA, USA) as previously described [43]. Soluble complexes were released from the thiol sepharose beads by reducing the E126C LF_N_-bead disulfide bond using 50 mM dithiothreitol (DTT) (Goldbio, St. Louis, MO, USA) in Assembly Buffer. To select for LF_N_-PA-nanodisc complexes, the released complexes were then incubated with Ni-NTA resin (Qiagen, Germantown, MD, USA). The His-tag on the MSP1D1 construct bound to the resin. Complexes were eluted from the Ni-NTA using 200 mM imidazole (Sigma-Aldrich, St. Louis, MO, USA) in Assembly Buffer. Assembled complexes were initially confirmed using negative-stain TEM.

Homogeneous 1LF_N_-PA pore complexes were produced using a modified protocol where E126C LF_N_ and PA were incubated in solution at a ratio of 1LF_N_:2PA prior to immobilization to reduce the number of complexes with multiple bound LF_N_. In this particular instance, affinity purification with Ni-NTA resin was omitted to minimize sample loss and homogeneous samples were still obtained.

### 5.3. Cryo-EM Sample Preparation and Data Collection

Cryo-EM samples were prepared within 10–30 min of elution. Three to four microliters (3–4 μL) of purified LF_N_-PA-nanodisc complexes were added to a glow-discharged holey carbon grid (Quantifoil R3/4 300 M Cu holey carbon) (Electron Microscopy, Sciences, Hatfield, PA, USA) and plunge frozen in liquid ethane using a Vitrobot (FEI, Hilsboro, OR, USA). Data were collected manually over the course of 10 sessions (8–10 h each) on a 2200FSC election microscope (JEOL, Peabody, MA, USA) at NCMI, Baylor College of Medicine. The microscope was equipped with an in-column energy filter (using a 20 eV slit) and operated at 200 kV acceleration voltage. Images were recorded on a Gatan 4k × 4k CCD camera using a 60,000× nominal magnification (1.81 Å/pixel) with an overall range of defocus values from one to three microns using a dose of approximately 20 e^−^/Å^2^. Approximately 650 individual micrographs were recorded. Homogeneous 1LF_N_-PA-nanodisc complexes were imaged and screened using a Tecnai F30 G2 twin transmission electron microscope (FEI, Hillsboro, OR, USA) at 200 kV at the University of Missouri Electron Microscopy Core Facility (EMC).

### 5.4. Image Analysis and 3D Reconstruction

The 650 raw micrographs obtained at Baylor were evaluated using EMAN2.1 [44]. At the early evaluation stage, around 250 of these micrographs were rejected due to either gross contamination or charging artifacts visible in the Fourier transforms. A total of 30,696 particles were manually boxed out using the e2boxer.py routine of EMAN2.1 with a box size of 224 × 224 pixels. The data evaluated with EMAN2.1 and RELION, showed a heterogeneous population of single, double, and triple LF_N_-bound PA. Due to this heterogeneity, it was difficult to use earlier versions of RELION with this smaller dataset to produce a model without imposing C7 symmetry. The approximately 30,000 particles were reevaluated using cryoSPARC (version 0.5). First, 2D class averaging was performed (Figure 5A). Bad classes were visually identified and discarded (e.g., unrecognizable densities, smaller than predicted density envelopes, etc.). Using the remaining 18,806 good particles, an *ab initio* reconstruction using the cryoSPARC SGD was carried out to computationally purify the dataset into subsets containing one, two, or three bound LF_N_. This computation was performed with the following settings: four groups, a group similarity factor of 0.2, and 10-fold the default iterations.

The SGD algorithm allows for *ab initio* structure determination that is insensitive to initial model inputs. An arbitrary computer-generated random initialization model improves over many noisy model iterations. Each step is based on the gradient of the approximated objective function obtained with a random selection of a small batch of initial particles. These approximate gradients do not exactly match the “overall optimization objective” (best *ab initio* model) but through multiple rounds, the derived models gradually approach this maximum. As stated by Punjani, Brubaker, and colleagues, “the success of SGD is commonly explained by the noisy sampling approximation allowing the algorithm to widely explore the space of all 3D maps to finally arrive near the correct structure” [28,29]. In contrast to using the entire dataset for initial model reconstruction, cryoSPARC samples random subsets of the images during its rapid iteration processes.

**Figure 11:**
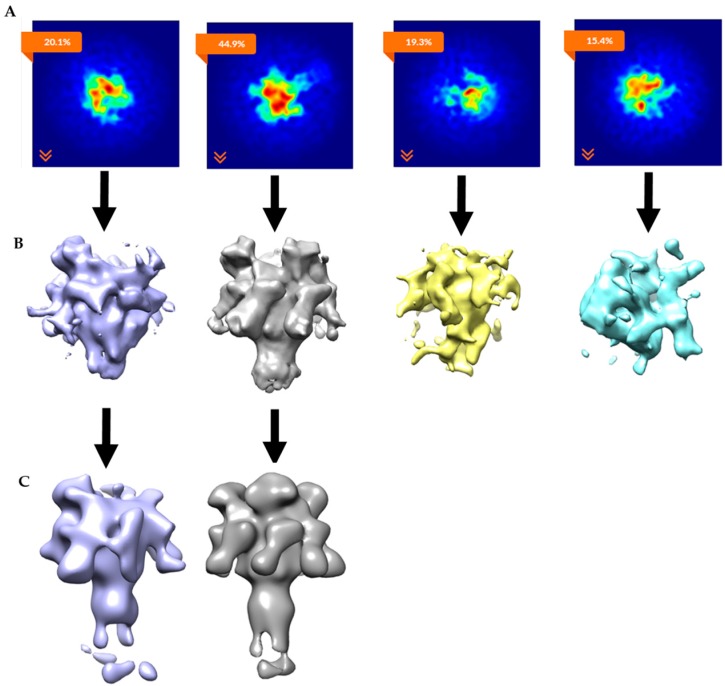
CryoSPARC data analyses parsed out heterogeneous LF_N_-PA-nanodiscs: (**A**) Image projection of heterogeneous *ab initio* reconstruction with four groups, the largest group, with 44.9% of particles, corresponds to 3LF_N_; (**B**) *ab initio* 3D models (side views); and (**C**) homogeneous refinements of *ab initio* group 1 and group 2. Group 2 refined to 18 Å model of 3LF_N_-PA from 4732 particles. Group 1 clearly shows missing density in the cap region and will need more particles to determine if this structure contains sub-saturated populations (i.e., one or two LF_N_ bound) of LF_N_ bound to the PA pore structure or that this group will split out further to separate one vs. two LF_N_-bound populations.

The *ab initio* model with three clearly-resolved LF_N_ densities possessed the largest percentage of particles (44.9%). The second most populated class (20.1%) appeared to contain one prominent LF_N_ density with the hint of a second bound LF_N_, but requires more particles in order to achieve definition (Figure 11B, column 1). After the *ab initio* model was generated, a homogeneous refinement with 100 additional passes using the branch-to-bound maximum likelihood optimization cryoSPARC algorithm. The final cryo-EM map resolution was estimated to be 17 Å based on Fourier Shell Correlation (FSC) with a cut off of 0.5 (Figure 5). The *ab initio* group 1 with the second highest percentage (20.1%) had one LF_N_ density at a lower volume threshold. However, further processing of the potential single bound LF_N_ revealed added density on the PA pore cap from a mixture of one and two LF_N_ populations (Figure 11C, column 1). More particles are needed to populate this distribution before definitive single or double LF_N_-bound structures can be obtained.

The cryoSPARC 3D reconstruction software tool (Structura Biotechnology, Toronto, ON, Canada) was run on a single workstation (Nova 2 Model: 2 × NVIDIA Titan Xp GPU, Intel Xeon E5-1630v4 (4-core 3.7 GHz CPU), 64 GB DDR4-2400 RAM, Intel 1.2 TB SATA solid state drive for runtime cache, and 4 × 4 TB Seagate SATA HDDs) purchased from Silicon Mechanics (Bothell, WA, USA) housed in the Fisher Laboratory. One of the main advantages of using cryoSPARC in combination with this computer system is the reduced computational time. What was once days or weeks in computational time is now only minutes or hours [29]. For example, as this paper was being written, the latest version of cryoSPARC was released (upgrade from 0.41 to 0.5). All Baylor collected data was reanalyzed with the newer version as a test for reproducibility in the span of 4 h (from reevaluating 2D classification, removing poor particles, etc.) where the final output *ab initio* models, reevaluated 2D class averages from separated populations and refined structures were reproduced using the single workstation described above. The use of SGD algorithms to generate *ab initio* models are now being beta tested or implemented in other software packages.

### 5.5. Molecular Dynamics Flexible Fitting of 3LF_N_-PA

A molecular model was fit into the cryo-EM density map using molecular dynamics flexible fitting (MDFF) methods [45] which apply an additional potential derived from the density map to the molecules. The starting molecular model was built by rigid docking three LF_N_ (PDB 3KWV) onto the PA pore cap (PDB 3J9C). The cryo-EM density map and initial molecular model were spatially aligned in Sculptor [46,47]. The density map was then converted from mrc to a situs file extension for compatibility with the Visual Molecular Dynamics (VMD) software suite. The atomic model and density map files were prepared for MDFF fitting in VMD by the typical MDFF tutorial progression [47,48]. The model was minimized for 2000 steps simulated for 50 ps at 300 K in vacuum. The grid-scaling factor, which controls the relative strength of the MDFF potential was set to 0.3. Figure 10 compares the 17 Å filtered MDFF structure with the 17 Å cryo-EM derived structure to show distinct similarities in surface topologies [46,48,49].
